# Astrocytes and human artificial blood-brain barrier models

**DOI:** 10.17305/bjbms.2021.6943

**Published:** 2022-04-01

**Authors:** Tanja Zidarič, Lidija Gradišnik, Tomaž Velnar

**Affiliations:** 1 Institute of Biomedical Sciences, Faculty of Medicine, University of Maribor, Maribor, Slovenia; 2 Department of Neurosurgery, University Medical Center Ljubljana, Ljubljana, Slovenia

**Keywords:** Astrocytes, blood-brain barrier, *in vitro* models, bioengineering

## Abstract

The blood-brain barrier (BBB) functions as a highly selective border of endothelial cells, protecting the central nervous system from potentially harmful substances by selectively controlling the entry of cells and molecules, including components of the immune system. To study the BBB properties, find suitable therapies, and identify new drug targets, there is a need to develop representative in vitro BBB models. In this article, we describe the astrocyte roles in the BBB functioning and human *in vitro* BBB models.

## INTRODUCTION

To move towards effective therapeutic solutions for neurodegenerative diseases, it is imperative to understand human brain physiology. Although animal models (rodents, primates, and worms) and experimental systems such as yeast have helped broaden our horizons about brain development, function, and disease, they cannot fully mimic the complexity of the human brain [1]. While relatively similar to those of other mammals, human brains have some species-specific adaptations. The cerebral cortex, which is associated with psychiatric disorders, is less complex in rodents than in primates and humans. In addition, human brains have a higher proportion and complexity of neocortical astrocytes than rodent brains [1,2]. Furthermore, different evolutionary pathways have led to differences in specific gene expression patterns, lipid profiles, or synaptic proteome composition between mammalian species, associated with interspecies differences in the distribution of drugs and endogenous substances in the human brain. Consequently, these interspecies differences are challenging for modeling complex neurodegenerative diseases and psychiatric disorders in animals, especially for drug and target discovery [1,3].

Most therapeutic agents are unable to cross the blood-brain barrier (BBB), which is a major hurdle to the successful treatment of central nervous system (CNS) diseases [4]. The strong barrier function of the BBB protects the CNS from potentially harmful substances by selectively controlling the entry of cells and molecules, including components of the immune system. As one of the major players in the immune surveillance of the CNS, disruption of the BBB results in extensive leukocyte infiltration and vasogenic edema, leading to various pathological conditions such as multiple sclerosis, encephalomyelitis, and stroke. In addition, significantly higher BBB permeability has been associated with mild cognitive impairment and early stages of Alzheimer’s disease. It is well known that the occurrence of brain tumors increases the permeability of the BBB, allowing macrophages and lymphocytes to heavily infiltrate brain tissue [5].

Considering this, there is a need for reliable models for fundamental academic and pharmaceutical research [5]. A major obstacle in brain drug development is the successful transition of therapy from *in vitro* models to preclinical animal studies and further to human clinical trials. For this reason, a more physiologically accurate *in vitro* model of the human BBB would be beneficial for finding candidate therapies and identifying new drug targets by studying multicellular interactions. To date, *in vitro* modeling of the BBB has focused primarily on two-dimensional (2D) transwell models. While these models can recapitulate many crucial aspects of drug transport across the BBB, their predictive power for brain drug uptake *in vivo* is limited [1,4,6]. The diversity of models and a further lack of standardization lead to variability between studies and make it difficult to compare data obtained with different models. BBB permeability is one of the parameters often inconsistently compared across studies, leading to inconsistent observations and misinterpretations. As a measure of the rate of drug delivery to the CNS (i.e., the rate at which drugs pass through the BBB), it must be considered separately from the extent (e.g., the ratio of drug concentration in brain and plasma) of drug equilibration through the BBB and brain distribution data to fully understand drug delivery to the brain and its implications for central drug action [7,8]. Recently, some attempts have been made to develop an *in vitro* BBB model that is more adaptable to current pharmacological developments for high-throughput drug screening and better correlate between *in vitro* and *in vivo* BBB permeability values [7,9,10]. Advances in biofabrication technology have paved the way to achieve higher physiological complexity of *in vitro* BBB models, improving their predictive power. This realization can already be seen with the creation of three-dimensional (3D) brain-like tissue structures that better replicate the human brain’s cellular composition, microenvironment, and architecture. The ever-growing field of biomedicine, which encompasses the latest discoveries in materials science, bioengineering technology, and cell culture has led to a variety of *in vitro* models of the BBB that primarily serve as mid- to high-throughput platforms in the early stages of drug development. Organoid technology or bioengineering strategies have been the main tools to design various 3D *in vitro* BBB models. Although they have only been in the scene for a few years, organoids have proven their merits in modeling human development, function, disease, and evolution. Their initial attempts at therapeutic applications in neurology, infectious diseases, and cancer biology have also been described. Since their formation and development are based on self-assembly, problems with reproducibility, heterogeneity, scalability, and necrosis continue to hinder their wider use.

On the other hand, bioengineering methods have made it possible to reconstruct the hierarchical structure of brain tissue through the precise spatial distribution of cell-containing hydrogels (so-called bioink). These approaches offer a higher degree of control and reproducibility, but often lack the natural microenvironment’s inherent complexity and physiological properties. Despite the ability to rapidly reconstruct the intrinsic components and structure(s) of the target tissue with high precision *in vitro*, 3D printing of BBB modeling is still in its infancy. Because there are no high-throughput 3D bioprinting BBB models available for research, this technology is not yet suitable for drug discovery and toxicology studies. The complexity of the tissue to be reproduced exponentially increases the complexity of the technical challenges to be overcome, including conjugation of support materials, cell types, and cell distribution, as well as delivery of the necessary biological factors to maintain cell viability and construction of the tissue scaffold itself [11]. More detailed explanations of 3D printed approaches to the construction of *in vitro* BBB models can be found in recent reviews [11-13]. Nonetheless, there is clear potential for combining these strategies to construct advanced 3D *in vitro* models of the human brain that address the current meagerness of treatment options and provide solutions for patients currently suffering from neurological disorders [1].

## NEUROVASCULAR UNIT AND BLOOD-BRAIN BARRIER

**FIGURE 1:**
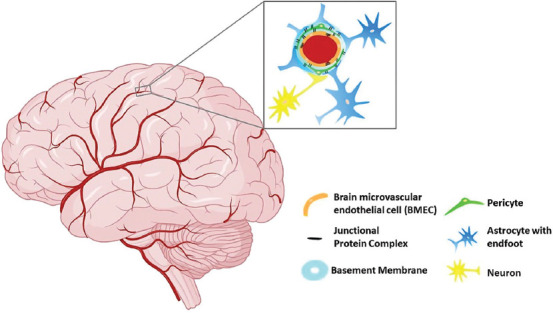
Schematic presentation of the NVU and BBB. The neural (astrocytes) and vascular components (BMECs, pericytes) of the NVU form the BBB. The basement membrane, which surrounds the BBB endothelium, encapsulates pericytes. NVU: Neurovascular unit; BBB: Blood-brain barrier; BMEC: Brain microvascular endothelial cell.

The main topic of neuroscience has traditionally been the peripheral nervous systems and their interactions with the glial cells that support their function. In recent years, the relationship between brain microvascular endothelial cells (BMECs), pericytes, vascular smooth muscle cells, glial cells (microglia, astrocytes, and oligodendrocytes), and neurons to form functional neurovascular units (NVU) between vascular districts and the CNS parenchyma has also gained extensive popularity. A NVU (Figure 1), the concept defined by Harder et al. [14], is a functional structure that connects the neural and vascular components of the brain, forming a BBB that maintains healthy brain physiology. In addition to the cells of the NVU, there is the non-cellular extracellular matrix (ECM) that provides structural support and biochemical cues to the NVU cells and enables cell adhesion and mechanical feedback between the cells and the extracellular environment. A specialized component of the NVU ECM is the basement membrane, which surrounds the endothelium of the BBB, encapsulates pericytes, and has different structural properties than the surrounding ECM of the parenchyma. The vasculature in the brain is critical for supplying oxygenated blood and nutrients to all parts of the brain and for removing waste products. Each component is closely and reciprocally interconnected, forming an anatomical and functional whole that results in a highly efficient cerebral blood flow regulation system [15,16]. The importance of specific interactions between the brain endothelium, astrocytes, and neurons within the NVU in regulating the BBB for maintaining healthy brain function has been highlighted by several groups [5,6,17].

The complex and highly controlled microenvironment is responsible for the normal functioning of the CNS. The molecular exchange between the blood and the neuronal tissue or its fluid spaces, and thus the brain’s homeostasis, is limited and essentially regulated by three biological barriers that allow undisturbed neuronal function within the parenchyma while ensuring immune surveillance borders of the CNS [18]. Each of these barriers is established by different cells:

1) The blood-cerebrospinal fluid (CSF) barrier, formed by the epithelial cells of the choroid plexus facing the CSF,
2) The avascular arachnoid barrier, which surrounds the CNS under the dura mater,
3) The BBB, formed by the BMECs [19-21].

These three biological interfaces form barrier layers between the CNS and the blood. However, because the BBB at the brain endothelium (i.e., localized at the endothelial cells of the CNS microvasculature) represents the first interface separating brain parenchyma from peripheral blood, it is consequently the most important [5,18]. The shortest diffusion distance to neurons makes it the leading site for exchanging molecules between the blood and the CNS [20]. It acts as a selective barrier for substances in the blood and separates the reservoir of neurotransmitters and neuroactive agents in the central and periphery nervous system to avoid crosstalk between these two systems. However, the most renowned role of the BBB is to protect the brain parenchyma from blood-borne agents and to prevent drugs and other exogenous substances from entering the CNS [21,22]. Overall, the BBB acts as a dynamic interface that regulates brain homeostasis and protects the CNS, responding to various physiological and pathological conditions [21].

### Structure

At its core, the BBB is a complex anatomic structure within the NVU at the level of the parenchymatous microvasculature of the brain, which includes capillaries, precapillary arterioles, and postcapillary venules. It is formed by BMECs. However, the properties of the BBB are not intrinsic to CNS microvascular endothelial cells but instead rely on the continuous interplay of the cellular and acellular elements of the NVU [18,23]. Most mammals and other organisms with a well-developed CNS align the brain (cerebral) capillaries that penetrate the brain and spinal cord [19,21]. Historically, the BBB was described as the layer of endothelial cells that form the capillary walls [24]. Unlike other vascular endothelial cells comprising of peripheral blood vessels, BMECs have distinctive morphological, structural, and functional features. These include:

- The expression of tight junctions (TJs), resulting in high transendothelial electrical resistance (TEER) and detained paracellular flux,
- The absence of fenestrations,
- The absence of pinocytic activity, and
- The expression of active transport mechanisms [19,21,23,25].

These features distinguish them from other vascular endothelia [19,21]. In addition, BMECs express specific enzymes and transporters that allow efficient transport of nutrients into the CNS and efflux of toxic metabolites from the CNS [23]. Interactions between BMECs, astrocytes, and pericytes facilitate the formation and maturation of TJs within the interendothelial space. Molecularly unique, complex, and continuous TJs include TJ proteins, adhesion junctions (AJs), junctional adhesion molecules (JAMs), and accessory proteins [22,23]. The TJs are critical for the specific functions of the BBB. They are responsible for the “physical barrier” of the BBB and contribute to regulating molecular transport properties. Among the various molecular transport pathways (paracellular transport, transcellular transport, transcytosis, and pumping), the TJs between adjacent BMECs ensure that most molecular traffic takes a transcellular route through the BBB rather than moving paracellularly through the junctions, as is the case in most endothelia [17,26].

In terms of molecular composition, TJs are composed of transmembrane proteins, cytoplasmic plaque proteins, signaling proteins, and adaptors that bind these complexes to the actin cytoskeleton. Transmembrane proteins (claudins, occludin, and JAMs) are thought to be the elements that form the barrier because they are composed of transmembrane, cytoplasmic, and extracellular domains. Claudins, occludin, and JAMs expressed in TJs are connected to the cytoskeleton via scaffolding proteins (zonula occludens [ZO] proteins) [26,27]. On the other hand, AJs are a prerequisite for the maturation and maintenance of TJs by establishing cell-cell contacts. Continuous crosstalk between AJs and TJs is necessary for the organization and maintenance of junctional complexes [27,28]. Despite these unique biochemical features, BBB endothelial cells share some characteristics with endothelial cells in peripheral microvascular beds. For example, all endothelial cells develop AJs and can express TJ proteins, but in the BBB, AJs are accompanied by complex and continuous TJ strands surrounding the entire circumference of BMECs [18,27].

As mentioned earlier, NVU contains the BBB endothelium, the endothelial basement membrane with a high number of embedded pericytes, and the glia limitans, consisting of the parenchymal basement membrane and astrocytic endfeet [5,18,19,21,22,27]. This complex structure is a highly coordinated system that dynamically regulates cerebral microvascular permeability, and maintenance of a functional NVU is a prerequisite for BBB function. For this reason, it provides a basis for understanding the development and physiology of the BBB, particularly the mechanisms underlying cerebral microvascular permeability. This is important for elucidating the influence of drugs and diseases on BBB permeability. Intracellular communication between components of the NVU regulates the maintenance of barrier properties and subsequently influences the permeability of the BBB [19,22,27].

BMECs form a selective barrier that covers the inner surface of cerebral capillaries. Therefore, they largely determine the permeability of the BBB to the vast majority of circulating substances. One of the most important features of BMECs is their ability to cross-talk with adjacent cells, AJs and TJs. In the former, the cadherin proteins are core AJ components associated with the cell cytoplasm in the intercellular cleft and mediate adhesion of BMECs to each other, initiate cell polarity, and partially regulate paracellular permeability. At the same time, TJs close the intercellular cleft by forming a multiprotein complex of transmembrane proteins (claudins, occludin, and JAMs) and cytoplasmic proteins (ZO-1, ZO-2, ZO-3, and cingulin) associated with the actin cytoskeleton. Loss of any of these proteins can severely compromise the integrity and functionality of the BBB. The TJs also form a fence around the cell, separating the luminal part from the basolateral region.

The restricted movement of small monovalent ions such as Na^+^ and Cl^−^ results in a much higher TEER for the brain endothelium *in vivo* than for peripheral capillaries (1000 Ω cm^2^ vs. 2-20 Ω cm^2^). Membrane surface charge, i.e., the cumulative effect of charged proteins, ions, and lipid headgroups on the membrane, is critical for homeostasis and protein targeting. The membranes of mammalian cells are generally known to be anionic. However, the membranes of BMEC exhibit a more anionic character than the membranes of vascular endothelial cells. They also show a preferential interaction with a peptide that combined cationic and lipophilic properties. At the BBB level, this particular feature of BMEC membranes contributes to the high selectivity of BBB. Moreover, it also answers the question of why lipophilic cationic molecules tend to interact with the BBB and explains the low adhesion of cells at the level of the brain [19,29].

For barrier selectivity, it is also important to highlight the importance of efflux carriers expressed by BMECs, most of which belong to the adenosine triphosphate-binding cassette transporter family (ABC). ABC transporters are highly expressed at the apical surface of BMECs and mediate the efflux of lipophilic compounds that would otherwise readily diffuse through the BBB. Among them, P-glycoprotein (P-gp) is one of the best characterized ABC transporters, as it is ubiquitously expressed and exhibits broad substrate specificity encompassing a variety of structurally distinct drugs from different pharmacological groups. These efflux systems may also have overlapping substrate affinities and actively pump compounds from BMECs back into the circulation, resulting in reduced CNS exposure. In addition, overexpression of P-gp has also been linked to the pathophysiology of various neurological diseases and the development of drug resistance.

Because they express a specialized set of drug-metabolizing enzymes such as alkaline phosphatase, γ-glutamyltranspeptidase, aromatic acid decarboxylase, and various cytochrome P450 (CYP) enzymes, BMECs also form a metabolic barrier that can metabolize numerous xenobiotics. It has been found that CYP mRNAs are present in various areas of the human brain that play a role in neurodegenerative diseases through drug and fatty acid metabolism. Since their expression is much higher in BMECs than in non-neuronal capillaries, they are considered as BBB markers [17,19,21].

BMEC properties determine BBB properties but depend on their communication with other NVU components. Extracellular vesicles (EVs) play a central role in communication between cells over short or long distances. EVs are a highly heterogeneous group of small cell-derived membranous particles containing various nucleic acids, proteins, and lipids. They have gained attention as important in intercellular communication between brain cells and several neurodegenerative diseases [30-32]. EVs can interact with endothelial cells, which are the brain’s first line of defense. Such interactions, mediated by surface markers, in some cases allow EVs to overcome the BBB or alter its properties [30]. EVs are also the pathway for NVU cells to respond to various environmental stimuli. The molecular signatures for brain endothelial cell-specific EVs have been identified under various biological conditions, providing a potential source of useful biomarkers and possibly novel receptors that should facilitate drug delivery [30,33] (reviewed in Reference [30]).

Apart from BMECs, pericytes are essential components of brain capillaries with varying abundance in different vascular beds. They are most abundant in the CNS, especially in the retina. They share a basement membrane with BMECs, and their close interconnection allows the exchange of ions, metabolites, second messengers, and RNAs between the two cell types [19,21,34]. They are involved in the stabilization of small vessel architecture, neovascularization, and angiogenesis [34,35]. Therefore, loss of pericytes leads to abnormal vascular morphogenesis, endothelial hyperplasia, and increased permeability in the brain [19,21]. Because they have similar contractile properties to smooth muscle cells, they can (to some extent) regulate capillary diameter and thus cerebral blood flow [21,36]. In addition, pericytes may exhibit phagocytic functions and help remove toxic metabolites [21,37], and have also been reported to possess multipotent stem cell capabilities [38]. The close association with BMECs enables pericytes to regulate specific BBB gene expression patterns in BMECs. Moreover, they induce polarization of astrocytic endfeet and coordinate bidirectional signaling between cells in the NVU. From a clinical point of view, brain pericytes may be directly or indirectly involved in various CNS pathologies, so they are increasingly considered as potential drug targets [19,34,35,39,40].

The complex structure of the BBB also includes other modular structures organized as gliovascular units. In this unit, the endfeet of astrocytes tightly envelop the pericytes and endothelial cell wall, releasing trophic factors essential for the induction and maintenance of the BBB. In addition, astrocytes are also involved in the control of cerebral vascular tone, synapse formation and function, as well as adult neurogenesis. Astrocytic endfeets or terminal processes cover a large portion of the basolateral surface of BMECs and play an essential role in regulating osmotic balance in the CNS [19,22,41,42]. Depending on their location and association with other cell types, they display 11 different phenotypes, 8 of which are involved in specific interactions with blood vessels. Their regional environment influences their phenotypic diversity in the brain, especially by dynamic changes in this environment during pathology, stress, or inflammation [43,44] (reviewed in References [43,44]). In addition, they upregulate many features of the BBB, resulting in tighter TJs, increased gene expression, and polarized distribution of transporters and specialized enzyme systems [19,22,41]. BMECs exhibit BBB properties even before astrocytic differentiation, demonstrating that astrocytes are not the sole basis for BBB formation. However, as highly fibrous structures between neurons and vessel walls, where they are coupled with neurons and BMECs, they are crucial in the metabolic activity of the BBB. The astrocytic surface expresses receptors for various neurotransmitters and neuromodulators required for neuronal signal transduction [22,45,46]. As part of the dynamic regulation of the neuronal system, astrocytes therefore play an important role in CNS inflammation in neurodegenerative diseases [21].

Microglia, cerebral perivascular macrophages, are the resident immunocompetent cells of the brain. They control innate and adaptive immune responses in the brain and modulate specific properties of the BBB by regulating TJs and thus paracellular permeability [19].

### Function

The BBB has several functions with key aspects of homeostasis [17,20], among others:

- Controls molecular traffic, repels toxins, leading to minimization of neuronal apoptosis,
- Contributes to ion homeostasis for optimal neuronal signaling,
- Provides a low-protein environment in the CNS, limiting proliferation, and preserving neuronal connectivity,
- Separates central and peripheral neurotransmitter pools, reduces crosstalk, and enables non-synaptic signaling in the CNS,
- Enables immune surveillance and response with minimal inflammation and cellular damage [20].

The BBB provides a controlled microenvironment through specific ion channels and transporters that keep ion composition optimal for neuronal function. This restricts ion and fluid movement between the blood and the brain. It also allows specific ion transporters and channels to regulate ion traffic to produce an interstitial cerebrospinal fluid (ISF) that is an optimal medium for neuronal function and synaptic signaling. In addition, it provides the brain with vital nutrients and mediates the efflux of many waste products [17,21,47]. A primary function of the NVU is to regulate transport and diffusion through the endothelial cells of the brain capillaries [22]. Small gaseous molecules such as O_2_ and CO_2_ can diffuse freely through the lipid membranes. The latter is also a portal of entry for small lipophilic agents, including drugs such as barbiturates and ethanol. As for small hydrophilic molecules, their transcellular traffic is regulated by the presence of specific transport systems at the luminal and abluminal membranes. This, in turn, provides a selective “transport barrier” that allows or facilitates the entry of needed nutrients and excludes or effluxes potentially harmful compounds. Finally, a combination of intracellular and extracellular enzymes provides what is known as a metabolic barrier. Ecto-enzymes such as peptidases and nucleosidases are able to metabolize peptides and ATP, respectively, while intracellular enzymes such as monoamine oxidase and cytochrome P450 can inactivate many neuroactive and toxic compounds [17].

The ISF of the brain is similar in composition to blood plasma, except that it contains a much lower protein content and lower K^+^ and Ca^2+^ concentrations but higher Mg^2+^ levels. Therefore, the BBB also protects the brain from fluctuations in ion composition after a meal or exercise and disrupts synaptic and axonal signaling [17,48]. The barrier helps to keep separate the pools of neurotransmitters and neuroactive substances that act centrally (in the CNS) and peripherally (in peripheral tissues and blood). This separation allows similar agents to be used in the two systems without “crosstalk.” The transfer of neurotransmitters from the brain to the blood depends primarily on Na^+^-coupled and Na^+^-independent amino acid transporters. Due to the sodium dependency and the substrate specificity of amino acids, the BBB limits the influx of some of them, including the neurotransmitters glutamate and glycine, while allowing many other essential amino acids to efflux [17,21,49]. The endothelium primarily regulates the brain microenvironment due to its large surface area (~20 m^2^ per 1.3 kg of brain) and short diffusion distance between neurons and capillaries. The epithelium of the choroid plexus (the blood-cerebrospinal fluid barrier and responsible for CSF production) is also involved in this process [17,50]. The continuous turnover and drainage of CSF and ISF by bulk flow contribute to the removal of larger molecules and metabolites of the brain, which further supports the homeostasis of the brain microenvironment [17,47].

The BBB allows low passive permeability for essential water-soluble nutrients and metabolites required by nervous tissue. In contrast, specific transport systems are expressed in the BBB to ensure an adequate supply of these substances for other nutrients that cannot pass. The selective and region-specific (luminal and abluminal surfaces of the endothelial cells) expression of these transporters gives the endothelium of the BBB its normal polarity [17,50,51].

Under physiological conditions, the BBB prevents many macromolecules from entering the brain through the normal paracellular or diffusion pathways. When these large serum proteins enter the brain through a damaged BBB, serious pathological consequences can occur [21,52]. There is a wide distribution of different activators for these proteins in the CNS. These include factor Xa, which converts prothrombin to thrombin, or tissue plasminogen activator, which further converts plasminogen to plasmin. Both resulting proteins (thrombin or plasmin) can bind to their receptors in brain tissue and initiate cascades that manifest in seizures, glial activation, glial cell division and scarring, and cell death. From this point of view, the BBB can be considered as a “gatekeeper” that allows only the beneficial substances to pass through [21]. Many potential neurotoxins circulate in our blood, including those from endogenous sources (metabolites or proteins) or exogenous (xenobiotics) ingested with food or otherwise acquired from the environment. Thus, the regulation of the uptake of various circulating substances is based on the needs of the CNS. When the adult CNS is damaged, it has the limited regenerative capacity, and fully differentiated neurons have minimal ability to divide and replace themselves under normal circumstances. In the healthy human brain, neurons die continuously from birth to the end of life, and neurogenesis is relatively low. That being said, any factor that accelerates the natural rate of cell death (e.g., increased access of neurotoxins to the brain) would lead to premature weakening [21,53].

### Role of astrocytes

Earlier studies suggested that the ability of BMECs to form a BBB was not intrinsic to these cells, but that the CNS environment with its glial cells induced this barrier property into the blood vessels. However, some researchers later contradicted the view that mature astrocytes play a significant role in the initial expression of the BBB, so the role of astrocytes remains enigmatic [41,54]. Regardless, astrocytes are considered an indispensable element of the NVU or the extended BBB. In the NVU, astrocytes are located between neurons and endothelial cells. The strategic position of astrocytes enables them to regulate cerebral blood flow to adapt to dynamic changes in neuronal metabolism and synaptic activity [55]. Since they are the most abundant cells in the mammalian CNS, many of their functions are well known. In fact, they are involved in various physiological and biochemical tasks, including:

1) Compartmentalization of the neural parenchyma, maintenance of ion homeostasis of the extracellular space;
2) pH regulation;
3) Uptake and processing of neurotransmitters by providing energy-rich substrates to the neuron;
4) Mediation of signals from neurons to the vasculature [21,42,54,56].

To meet the metabolic requirements (oxygen and glucose supply) for optimal brain function, both BMECs and astrocytes participate in this process. Astrocytes surround the CNS capillaries and establish a tight interaction that controls blood flow in the CNS and is known as neurovascular coupling [21,22,42,57,58]. Neurotransmitters released by astrocytes can adjust signaling in neurovascular coupling, allowing glial cells not only to control BBB properties but also to regulate blood flow [42,58]. For example, glutamate-mediated signals can modulate blood flow in response to local oxygen concentration by releasing nitric oxide from neurons and arachidonic acid derivatives from astrocytes [58,59].

As aforementioned, astrocytes are characterized by a great deal of heterogeneity based on their location and their association with other cell types. Astrocytes from different anatomical regions of the CNS express different amounts and types of ion channels that influence their electrophysiological properties, including their resting membrane potentials. This high degree of heterogeneity within the astroglial population defines the unique structure of the CNS and may reflect distinct molecular and functional properties of the BBB [58,60]. At the level of the BBB, there are two basement membranes that separate the endothelium from the astrocytes: the endothelial basement membrane and the parenchymal or astroglial basement membrane. Through the basement membranes, the interplay between components of the ECM and matrix adhesion receptors achieves appropriate cross-talk between the cells and their microenvironment. In addition, components of the ECM have the ability to bind to endothelial cell receptors, contributing to the BBB phenotype [17,19,22,41,58,61].

Astrocytic endfeet regulates CNS homeostasis through increasing intracellular Ca^2+^ levels in endfeet [42,62,63]. This complex gliovascular system has a high density of specialized molecules, including purinergic P2Y receptors, the potassium channel Kir4.1, and the water channel protein aquaporin-4 (AQP4), suggesting a key role in gliovascular signaling and in regulating water and electrolyte metabolism in the brain under normal and pathological conditions [21,64-66]. In addition, astrocytes are also instrumental in the formation of other features of the BBB, such as tighter TJs, specialized enzyme systems, and polarized localization of transporters (glucose transporter 1 [GLUT1] and P-gp) [41,42,58,62,63]. Astrocytes communicate with each other through gap junctions known as inter-astroglial gap junctions, which form a functional syncitium characterized by a well-coordinated response to stimuli. These inter-astroglial gap junctions transmit astrocytic mechanisms that modulate vasodilation and vasoconstriction [42,58,62,63].

The interactions between astrocytes, BBB and BMECs are also important for the development and maintenance of BBB properties. Astrocytes secrete growth factors such as vascular endothelial growth factor (VEGF), glial cell line-derived neurotrophic factor, basic fibroblast growth factor, and angiopoietin-1, which regulate BMEC function and different BBB features during development and adulthood [17,24,41,42,58]. However, there is an ongoing debate about the factors involved in this differentiation. There is agreement that several factors, some of which are soluble and some of which depend on cell-cell contact, participate in these mechanisms [41,67]. In addition, BMECs also have the ability to regulate astrocyte phenotype by secreting growth factors (leukemia inhibitory factor). Moreover, astrocytes, together with BMECs and pericytes as part of the ECM structure, further influence the differentiation of BMECs [41].

## ENGINEERING THE BLOOD-BRAIN BARRIER

Recent progress in stem cell technology, tissue engineering, and microfluidics has led to a rapid increase in the complexity of *in vitro* models of the BBB. Since animal models do not always recapitulate human physiology or disease, *in vitro* models can bridge the gap between human physiology and *in vivo* models. In addition, human astrocytes are structurally more complex, larger, and conduct Ca^2+^ signals much faster than rodent astrocytes [60,68]. In fact, the value of BBB models in basic and translational research depends on the ability to recapitulate *in vivo* and *ex vivo* studies. The accuracy of the model is usually determined by the purpose and the processes being studied. Simplified models will naturally recapitulate fewer features of the BBB. On the other hand, more complex models can be used to mimic more properties, but this is usually accompanied by lower throughput. Regardless of the complexity of the BBB model, the important thing is to establish physiological relevance. Paradoxically, the limited knowledge of BBB structure and function comes from *in vitro* studies, which poses a major challenge for establishing criteria for validating *in vitro* BBB models. Depending on the purpose of the *in vitro* model, the specific criteria may vary. The following are commonly used criteria for human BBB tissue engineering that relate to structure, microenvironment (ECM), barrier function (TEER and permeability), cell function (expression of BBB markers), and co-culture with other cell types (astrocytes and pericytes) [69], as well as other criteria summarized in a review by De Stefano et al. [68].

### Extracellular matrix

In modeling the BBB, the role of the ECM cannot be ignored. In addition to the various cell types (which account for 70-85% of brain volume) within the BBB, the ECM serves as an anchor for the endothelium through the interaction of endothelial integrin receptors and matrix proteins such as laminin [24,42,70]. It is characterized by an interconnected network of 50-100 nm pores that serve as reservoirs for ions and transport pathways. The ECM in the brain is primarily composed of hyaluronic acid (HA), lecticans, hyaluronan and proteoglycan link proteins, and tenascins, which cross-link the HA to form a 3D network. All of these components are important for maintaining paracellular diffusion in the BBB [24,42,71,72]. Moreover, laminin is present in small amounts in the developing brain and injured adult brain [73], whereas many common ECM proteins, such as fibronectin and collagen, are not present in the brain [24,68,71]. On the contrary, fibronectin and IV type collagen together with laminin form the basement membrane surrounding BMECs and pericytes [24]. In this context, fibrin or collagen with its derivatives are often used as hydrogels to promote cell adhesion and proliferation [74-76]. Overall, effective human BBB tissue engineering models should either include large numbers of neurons, astrocytes, and other glial cells, or use a passive ECM-based material that provides structural support for the BBB microvessels [68].

### Expression of blood-brain barrier markers

Commonly used markers to characterize *in vitro* BBB models are the expression of TJs and transport systems [68,77]. While many cell types express TJs, claudin-5 is specific to BMEC and particularly enriched in cerebrovasculature [68,78]. In addition, the localization of claudin-5, occludin, JAMs, and ZO-1 is often a criterion for validating cell sources for *in vitro* models [68,77,79-82]. The general method for visualizing these proteins at cell-cell junctions is immunocytochemistry. In physiological environments, TJ strands should be seen flocculently, whereas in pathological or nonphysiological conditions, they are discontinuous or show intracellular localization [68,82-85].

Moreover, the brain endothelium is rich in nutrient and efflux transporter systems. Since GLUT1 is highly expressed only in BMECs, it is a common biomarker for brain microvessels and capillaries. The expression of GLUT1 is critical for the transport of glucose to fulfill the high metabolic requirements of the brain. The other appropriate markers for the BBB are the primary efflux transporters P-gp and breast cancer resistance protein (BCRP) [21,82]. These efflux systems have overlapping substrate affinities that actively pump compounds from endothelial cells into the bloodstream [21]. They are highly expressed in the luminal membrane of the BBB [21,86], and recent studies report some cooperativity of action [87] and substrate overlap [88]. Therefore, expression of key BBB-related genes (P-gp and BCRP) confirms transporter-substrate functionality, an important feature of BBB barrier permeability to potential drug molecules, supporting the formation of a functional BBB *in vitro* [82,89].

#### Transendothelial electrical resistance

In addition to molecular profiling, actual permeability measurement is critical for establishing thresholds and evaluating *in vitro* models. The most common method for assessing the permeability of any endothelial barrier is measuring TEER, also referred to as transepithelial electrical resistance. TEER is considered to be an excellent indicator of barrier integrity because electrical impedance across an epithelial or endothelial barrier depends on the formation of robust TJs between adjacent cells [90-92]. TEER is commonly used for traditional transwell BBB models. Recently, it has been increasingly applied in the emerging “organs-on-chip” technology [93,94]. However, TEER is difficult to measure in tissue-engineered microvessels (capillaries) because of the low resistance between the lumen and the surrounding matrix at the inlet and outlet. To overcome this problem, one way is to measure both TEER and the permeability of a small molecule (Lucifer yellow) in a transwell assay (a 2D system) and the permeability of the small molecule in 3D microvessels (a 3D system) in the same experiment. If the permeability of the small molecule in the 2D and 3D systems is the same and the TEER value in the 2D system is in the physiological range, the tissue engineering model possesses physiological TEER. However, this method should be used with caution. Although TEER is approximately inversely proportional to permeability, the relationship is not linear and depends on the endothelial cell type and solute [68,95,96]. Moreover, since there are no TEER values from human brain capillaries, the standard values of TEER are obtained from anesthetized rats and are often misquoted [97,98]. Indeed, these standard TEER values were set up on the fact that the arterial capillaries of the rat reach 6000 Ω cm^2^ and the venous vessels reach 5500 Ω cm^2^ [98]. As it turned out later, these values were the misinterpreted data. Although most published human *in vitro* BBB models are characterized by lower *in vivo* like values of TEER [26], there have been reports of human *in vitro* BBB models reaching TEER values around 5000 Ω cm^2^ [97,99]. In organ-on-chip systems, the microenvironment with microfluidic channels supporting cell growth and differentiation makes access to the cell layer difficult, making continuous TEER measurement to monitor permeability changes challenging [90]. Nevertheless, the approach of TEER, based on the principle of measuring electrical resistance through a cellular barrier, has proven to be a highly sensitive and reliable method for confirming the integrity and permeability of *in vitro* barrier models. Due to its non-invasive nature and the advantage of continuously monitoring living cells during their different growth and differentiation stages, it is widely accepted as a standard validation tool [90,100,101].

#### Permeability

Similar to TEER values, defining quantitative criteria for barrier function are challenging because almost all quantitative *in vivo* data come from animal models. The *in vivo* permeability of the BBB to various substances was determined using different experimental protocols in animal models [68,102-104]. To date, there is no single available marker that meets the following requirements: it must be metabolically inert, non-toxic at the administered dose, not bound to other molecules (proteins) in plasma or tissues, present in a variety of molecular sizes, visible to the naked eye to electron microscopy, and reliably quantifiable [21]. Consequently, there are discrepancies in animal studies that depend on the molecular weight of the solvents used to visualize the brain. For example, the permeability of the larger dextran at 10 kDa is 5 times lower than that of fluorescein (376 Da) [21,68,77]. Therefore, it has been suggested that low molecular weight markers (<400 Da), such as fluorescein, can be used to quantify subtle impairment of the BBB, because even a minor degree of barrier damage is likely to have a noticeable effect on its permeability that cannot be quantified with larger molecular weight markers [21,105]. If the BBB is damaged and loses its integrity, the likelihood of high molecular weight markers such as dextran entering the brain increases, and this could be considered as an option to assess the integrity and permeability of the BBB. Therefore, a combination of different markers is currently the most reliable approach to adequately assess the barrier integrity of the BBB [21].

*In vitro* BBB models generally do not achieve physiological permeability, which is evident in a limited number of permeability studies [68]. In one such study, tissue engineered BBB microvessel networks composed of primary human BMECs (hBMECs), astrocytes, and pericytes exhibited high permeability to a large molecule, which was inconsistent with the barrier function of the BBB *in vivo*. The observed high permeability was probably related to low TEER values (40-50 Ω cm^2^) of primary hBMECs [76]. In another study using induced pluripotent stem cell (iPSC)-derived hBMECs, the reported TEER values (>1500 Ω cm^2^) were within the physiological range. These results have supported the hypothesis that physiological TEER values are associated with negligible paracellular transport and permeabilities, which, in turn, are related to transcellular transport alone [106].

The impermeability of the human BBB with respect to its specificities is difficult to assess. Therefore, an analysis using different molecules is required to determine the characteristics of permeability. This, in turn, provides the guideline to replicate the barrier function in a representative BBB model more accurately. These permeability studies should include molecules such as:

- Transferrin family proteins, which are known to cross the BBB unlike other molecules of similar size;
- Albumin, which has a CSF to serum ratio that is associated with BBB integrity;
- Caffeine and nicotine, which are small molecules that can easily cross the BBB; and
- Drugs that have successfully crossed the rodent BBB in preclinical trials but have been unable to cross the human BBB; such drugs may be used as standards for human-specific impermeability [97].

Since these features characterize the human BBB, it is recommended that to use them to evaluate the accuracy of an *in vitro* barrier compared to an *in vivo* barrier [97]. In this context, Wang et al. [99] measured the selective conversion ability of cimetidine and caffeine, two molecules that can pass through the BBB. Because they used primary rat astrocytes, which are generally smaller and less complex than their human counterparts, they could not truly replicate the barrier function of the *in vitro* BBB model. Therefore, more examples of selective permeability and impermeability are needed for the development of future models [97]. Moreover, these studies should be designed from the perspective of the human BBB, which is more complex than that of other species.

### Astrocytes, pericytes and barrier function

As it appears, astrocytes, astrocyte extract, or pericytes contribute to the increased levels of TEER of endothelial monolayers *in vitro*, suggesting the role of astrocytes and pericytes in the upregulation and maintenance of barrier function *in vivo* [107,108]. However, the final TEER values often did not reach physiological values (1500-8000 Ω cm^2^), raising some doubts about their involvement. For example, monolayers of iPSC-derived hBMECs TEER have values in the physiological range, especially when cultured with retinoic acid [79,80], indicating that astrocytes and pericytes are not *per se* essential for achieving physiological TEER values in *in vitro* models *per se*. Because retinoic acid is secreted by radial glia, it may directly influence developing endothelial cells during differentiation of the BMECs to upregulate BBB properties, and it could indirectly promote BBB differentiation by inducing changes in neural cells. Alternatively, it may act through a combination of these mechanisms [79]. On the other hand, monolayers from iPSC-derived hBMECs cultured with astrocytes and/or pericytes under subphysiological conditions approached [109,110] or reached physiological TEER values [79,99]. Given these results, astrocytes and pericytes, although not directly involved in the establishment of barrier function, may indirectly help by secreting factors that promote BBB recovery or repair [68].

## *IN VITRO* HUMAN BLOOD-BRAIN BARRIER MODELS

When developing an *in vitro* tissue model, it should be thought of as a living structure. This also applies to *in vitro* BBB models due to the important role of the BBB in CNS homeostasis [97,111]. The development of *in vitro* BBB models has been driven primarily by the desire to understand the function of the BBB during development, health, and disease. Furthermore, because the BBB excludes the vast majority of small molecules, proteins, and gene therapeutics [111-113], *in vitro* BBB models are often used as screening platforms for the development of neurological agents [113].

Innovations in cell culture methods, biofabrication technologies, and biomaterials are driving rapid progress in *in vitro* modeling of the human BBB [111]. Although it is known that no simple *in vitro* model can mimic all functionalities of the BBB, there is a consensus that the model must have at least the most relevant features of the BBB that do not conflict with the particular study objective [19]. Furthermore, it is essential to consider the choice of cell types in order to closely mimic the physiological characteristics and response of BBB *in situ*. In general, among the various cell types (especially in brain microvascular endothelium), primary cells predominantly provide the best approximation to their natural counterpart. However, they are associated with the limited number of passages in culture before use and the purity of the original batch. Specifically, primary human cells are restricted in availability and generally depend on clinical samples of brain tissue. Cells isolated from resection of human brain tissue are likely to be associated with brain dysfunction. On the other hand, commercial sources of human primary cells, especially BMECs, are generally fetal primary cells that may not have the full differentiation spectrum of a mature BBB counterpart.

Commonly used primary cells are also of animal origin (usually rodents). Similar to human primary cells, animal-derived primary cells tend to differentiate quite rapidly *in vitro* and may respond differently to specific assays with respect to pharmacoresistance [11]. Several issues have limited the general use of BBB models based on primary hBMECs and immortalized human cells. These include loss of phenotype of hBMECs during cell culture, lack of important TJs, and low TEER levels in human cell lines. Recently, new human BBB models based on stem cells have been developed to circumvent these problems. Human pluripotent or multipotent stem cells are a promising source of cellular components for modeling *in vitro* human BBB models because these cells can differentiate into BMECs (although this ability depends on the origin of the stem cells). They can give rise to significant numbers of BBB cells and can be used to model BBB pathologies [114,115] (reviewed in References [114, 115]).

In the context of reconstructing the complete BBB, an ideal platform would provide physiological levels of shear stress (oriented hemodynamic forces) [116,117]. Shear stress plays a crucial role in promoting the differentiation of vascular endothelial cells into a true BBB phenotype by modulating the expression of TJs and transporters. In terms of morphology, BMECs exposed to physiological shear stress become flatter and larger, and show a marked increase in endocytotic vesicles, microfilaments, and clathrin-coated pits, thereby more closely resembling the BBB phenotype *in vivo* [25,118]. Circulating blood flow in the brain generates fluid shear stress, which acts on the vascular endothelium and is transmitted to the neurons and glial cells around the brain capillaries. The range of shear stress is 10-70 dyn/cm^2^ in the arterial circulation and 2.8-95.5 dyn/cm^2^ in the capillary circulation [25,119,120]. The wide range of shear stress in capillaries is partly due to neurovascular coupling and the wide range of metabolic demands. As in the human cerebrovascular network, shear stress in capillary vessels ranges from 5 to 23 dyn/cm^2^ [25,121,122]. However, reported values of applied shear stress in *in vitro* studies vary considerably because of inconsistent established *in vitro* models of BBB and different cell sources [25]. In addition, incorporating shear stress would also facilitate the correct spatial organization of NVU components so that they could form realistic cell-cell interactions and basement membranes [116].

In *in vitro* BBB models, cells are cultured in contact with different biomaterials that can fully or partially mimic the properties of the basement membrane (reviewed in Reference [117]). Due to their different physicochemical properties, these materials may differentially affect BBB formation *in vitro*. Synthetic materials used to culture BMECs are usually stiff (elastic modulus in the range of MPa to GPa) and either solid or porous (semipermeable membranes). These surfaces provide mechanical support for cell growth, proliferation, and migration in the monolayer configuration [11,117]. The substrates can be easily modified by absorbing protein surface coatings to facilitate cell adhesion and spreading [117]. For example, collagen IV and laminin proteins enhanced BMEC adhesion and proliferation and subsequent formation of an endothelial monolayer [123,124]. Various materials used to make porous membranes include polycarbonate (PC), poly(ε-caprolactone), polydimethylsiloxane, polyester, polyethylene terephthalate (PET), silicon nitride, and silicon dioxide [74,117]. Commercially available inserts made of PC or PET membranes have limited ability to mimic the natural basement membrane accurately. Because they are thicker, less porous, permanent, and made of foreign materials, they result in decreased cell adhesion, decreased cell-cell contact, decreased diffusion of soluble signaling molecules, and unnatural substrate interactions. Nevertheless, they proved their merit in a plethora of biological *in vitro* studies [74]. Appropriate membrane pore size may allow cell migration and limited physical cell-cell contact [16]. For endothelial cell cultivation, porous membranes with a pore diameter of 0.4 mm were preferred because larger diameters resulted in cell migration through the membrane [117,125].

**FIGURE 2:**
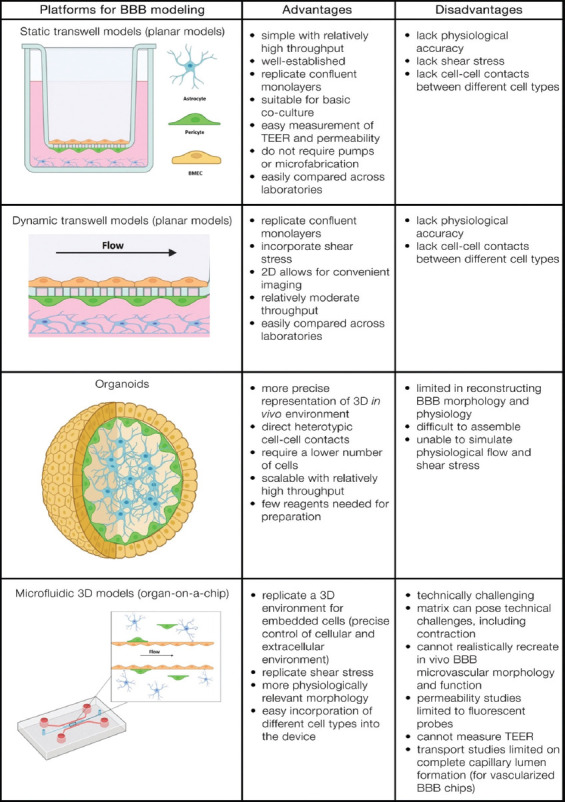
Platforms for engineering human BBB models (adapted from ref. [6, 116, 126]). BBB: Blood-brain barrier; TEER: Transendothelial electrical resistance.

Several *in vitro* models of the BBB (Figure 2) [126] have been developed that exhibit physiologically relevant features, but none of them has been adopted by the pharmaceutical industry as the “gold standard.” Therefore, the development of more reliable models to test the permeation of the BBB remains a current challenge. The most widely used and commercially available platform is the transwell chamber for assessing BBB integrity, barrier properties, permeability, activation, and inflammatory cell migration. In addition, a better understanding of BBB biology, together with recent advances in biotechnology and materials science, has enabled the development of innovative and highly integrated “quasi-physiological” *in vitro* BBB systems that aim to meet other criteria [6,19,111,113,116].

### Transwell models (planar models)

**FIGURE 3:**
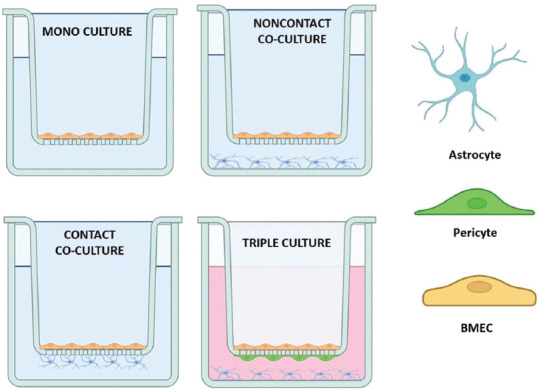
Commonly used configurations of transwell BBB models. Monoculture: BMECs are grown on the upper surface of the permeable support. Noncontact co-culture: BMECs are seeded on the upper surface of the support, while astrocytes (or other cell types) are seeded at the bottom of the culture well. Contact co-culture: BMECs are seeded on the top of the support and astrocytes (or other cell types) are seeded on the bottom of the support. Triple culture: In its most common form BMECs are seeded on the top of the support, pericytes seeded on the bottom surface, and astrocytes seeded on the bottom of the culture wells. BBB: Blood-brain barrier; BMECs: Brain microvascular endothelial cells.

Transwell models are described as simple layered structures that use novel cell sources and integrated assessment techniques. Commonly used configurations for transwell models are presented in Table 1 and Figure 3. They usually consist of endothelial cells (in mono or co-culture) cultured on an ECM-coated permeable membrane of a cell culture insert, which is often suspended in one well of a 12- or 24-well plate [77,113,116]. In the monoculture where no other cell types of NVU are present, the astrocyte-conditioned medium can be added to promote the proliferation and differentiation of BMECs [77].

**TABLE 1:**
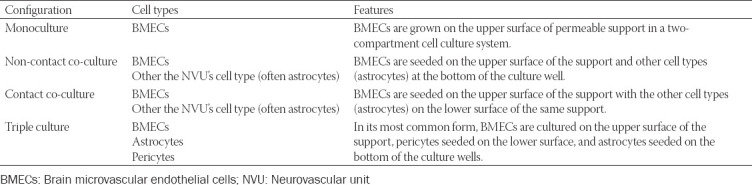
Common configurations of transwell models [77]

Despite the fact that their simplicity ignores vessel formation, it favors the model’s performance in other directions by reducing the number of variables under consideration and providing a more comprehensive characterization [116]. For permeability screening, molecules or cells can be added to the culture medium in the insert (apical or “blood side”) and their accumulation at the bottom (basolateral or “brain side”) of the well evaluated over time or vice versa. They also allow rapid and non-destructive quantification of barrier integrity through TEER [113]. Even though TEER has some limitations, it is accepted as a standardizable measure that allows easy comparison of permeability. In this respect, it can be used as a measure for models that aim to maximize the barrier function of the BBB [116]. However, the lack of fluid flow combined with the relatively large volume of the medium may attenuate the effect of cell-cell signaling by soluble factors. Another drawback of these models is the permeability of the membrane. This prevents substantial contact between BMECs and other NVU cell types in a transwell, which hinders cross-talk between cells [113].

Because of their simplicity, these planar (monoculture) platforms can also be adapted to create multicellular BBB models with the flexibility of positioning different cell types depending on the intended application of the model. It has been demonstrated that co-culturing and tri-culturing of astrocytes and/or pericytes with endothelial cells significantly increases the tightness of endothelial monolayers [127,128]. Intercellular adhesion between astrocytes in the BBB was observed in the form of AJs. The presence of glia and the establishment of glia-endothelial interactions have been shown to increase the expression of transporters such as P-gp and TJs and to help induce a phenotype more similar to that found *in vivo* [128]. Co-culture models can also be developed using pericytes [108] instead of astrocytes or neurons [129]. For example, NVU cell types can be cultured on the bottom of the well, allowing the exchange of soluble factors with BMECs cultured on the insert [77,113]. This non-contact co-culture allows for the induction of the endothelium by diffusible factors released from supporting cells (astrocytes or pericytes) at the bottom of the well [77,130]. This configuration can lead to a robust increase in TEER up to 5000 Ω cm^2^ [79]. It is more or less common practice that BMECs are cultured on the top of the porous membrane while supporting cells such as astrocytes and pericytes are grown on the bottom of the well [6,113]. This constellation is sometimes referred to as “contact” co-culture [77,130], even though the membrane prevents *in vivo*-like cell-cell contact [113]. In this way, the spatial separation of each cell type can be maintained for subsequent molecular analysis [6,113]. However, the disadvantage of this setting is that the two cell types cannot be readily separated in experimental protocols using Western blotting or transport and accumulation studies [77]. Given the importance of NVU cells in the development and maintenance of the BBB and the coordinated response to the pathological environment, *in vitro* models of the BBB should consider incorporating all critical cellular components (BMECs, astrocytes, and pericytes) to enhance the physiological relevance [77,127,130,131]. The beneficial effect of triple co-cultures and mixed co-cultures has been shown in creating a more efficient *in vitro* BBB model in terms of high TEER [108,127]. In particular, astrocytes induced a tighter barrier than pericytes. Pericytes, on the other hand, contributed to improved TJ formation when co-cultured with endothelial cells [127]. Overall, all research results agree that the model must closely mimic the situation *in vivo*, i.e., astrocytes and endothelial cells must be in close proximity (cell-cell contact) [127,132,133].

Despite the technical simplicity of a static planar model assembly, most experiments are based on the cultivation of rodent astrocytes and endothelial cells on transwell membranes [97,134]. So far, the only static planar human BBB model has been developed in the form of a bilayer co-culture of human astrocytes and endothelial cells derived from human iPSCs (hiPSCs) seeded onto an electrospun poly(lactic-co-glycolic acid) mesh. *In vitro* studies showed that electrospun scaffolds can reduce astrocytic reactivity and recapitulate the *in vivo* astrocyte phenotype *in vitro* better than 2D flat surface controls. The positive effect on astrocyte response is even greater when the electrospun fibers are arranged in a 3D architecture. Moreover, these scaffolds can be further improved by coating them with ECM proteins to improve TEER values, reduce permeability, and allow less separation between cells compared to standard polyethylene PET inserts [74,134-137]. The developed human BBB model was characterized by significant barrier integrity with the expression of TJ proteins and higher TEER value compared to electrospun mesh-based counterparts. These superior properties are due to the co-culture of hiPSC-derived astrocytes and endothelial cells [134]. Nevertheless, this model is not an improvement as the TEER values were higher than other planar models and known *in vivo* values [97,134]. This inadequate performance is likely due to the lack of shear stress experienced by endothelial cells under static conditions, as this is a property known to modulate TJ formation [97].

Furthermore, differentiation of human pluripotent stem cells into BMCEs may lack the functional properties of native hBMECs, which may hinder the development of a robust and physiologically relevant human *in vitro* BBB model for functional studies and drug discovery [138]. Recently, Rizzi et al. [131] developed an *in vitro* microsystem with triple culture configuration to predict brain penetration in preclinical assays. They presented a microphysiological system for modeling the BBB using only human cells, including brain-like endothelial cells, pericytes, and astrocytes. The applicability of the model was demonstrated using transport experiments with nanogels, which showed the possibility of performing transport experiments with a multicellular system. A limitation of the model was the lack of shear stress, which has been shown to affect endothelial cell differentiation and TJ protein expression. Nevertheless, the authors demonstrated that they could reproduce the architecture of the human BBB *in vitro* [131].

Most dynamic planar models of the BBB extend the static 2D transwell-based approach by incorporating a 10 μm thick transwell membrane into a microfluidic device. In a variant of the membrane-based microfluidic models, an ECM can be placed in the channel below the porous membrane, allowing the co-culture of other cell types in a 3D matrix. Although these models still have a planar geometry and a porous membrane that prevents full cell-cell contact, the introduction of a shear stress component contributes to more physiological conditions. Thus, these models are closer to the microenvironment of the BBB and allow for more advanced *in vitro* studies of drug permeability, where the effects on neurons could also be studied [116]. Both variants (with and without an ECM component) of 2D dynamic models can facilitate the application of shear stress through the flow of the medium, mimicking the effect of blood flow *in vivo* [6,113]. These microfluidic devices are often similar in design to a standard transwell in which BMECs are cultured in 2D, with a second compartment often containing supporting cell types. Flow is usually connected to the upper chamber to expose BMECs to shear stress, but may also be connected to the basolateral chamber depending on the device design. In addition, shear flow over the endothelial cells prevents the formation of an unstirred interface directly over the endothelial cells. Although this is often combated in conventional transwells by placing the plate on a rotator, this is an easily overlooked step that can lead to inaccuracies in measured transport [6].

Permeability measurements can be made by adding small molecules to the culture medium, and TEER can be evaluated with integrated electrodes [99,116,139]. These dynamic planar models are commonly used for low-to-moderate throughput screening applications (2-10 compounds of interest) that require both shear stress and the basic BBB properties of classical transwells models [6,139]. Consequently, they have been widely used to measure solute permeability, barrier integrity, and cytotoxicity, with test structures similar to those of the conventional transwell model. In addition, they have served as a tool to investigate the effects of NVU cell co-culture, medium composition, inflammation, and other physical parameters (cell morphology or turnover) [6,99,140]. Large biopharmaceuticals, such as therapeutic antibodies, have also been tested to investigate their ability to penetrate the BBB, which is often a tremendous challenge and limits efficient delivery to the brain. For this purpose, Wevers et al. [141] successfully integrated a human microfluidic BBB model in a plate-based high-throughput format with drug screening capability. The model included human cell lines of BMECs, astrocytes, and pericytes. The most important innovation was the vascularization of the model. A perfused vessel of BMECs was grown on a patterned ECM gel, with astrocytes and pericytes added to the other side of the gel to complete the BBB-on-a-chip model. The perfused BBB-on-a-chip model showed the presence of adherents and TJs. In addition, it showed sufficient barrier function to study the passage of large molecules and is sensitive to differences in antibody penetration, which could support the discovery and development of BBB shuttle technologies [141]. In another study, Rizzi et al. [131] developed triple culture *in vitro* microsystem to predict brain penetration in the frame of preclinical assays. They presented a microphysiological system to model the BBB using solely human cells, including brain-like endothelial cells, pericytes, and astrocytes.

2D shear devices allow many of the same assays that can be performed in traditional planar models. However, the addition of shear stress provides further insight into the transport mechanisms that are affected by shear stress and can be more easily imaged with live-cell microscopy. On the other hand, they have lower throughput than conventional transwells. Although they are gradually becoming more common as a standard research tool, there is still little standardization and platform design varies widely [6].

### Organoids/spheroids

In the most commonly used transwell system, BMECs are cultured on the top side of the transwell insert, whereas astrocytes and pericytes are cultured on the bottom side of the setup, which poses a challenge to obtain reproducible BBB properties and functions [142]. Self-assembled cell aggregates consisting of BMECs, astrocytes, and pericytes are emerging as a potential alternative to transwell and microfluidic models for certain applications. Unlike transwell models, these organoids allow direct contact between different NVU cell types and yield an endothelial monolayer for permeability studies [97,113]. Consequently, they can generate many features of the BBB, including the expression of TJs, molecular transporters, and drug efflux pumps, and thus can be used to model drug transport across the BBB [142]. Furthermore, additional properties of the BBB can be elucidated by producing organoids with all cell types present in the cortex in a ratio similar to normal tissue. Therefore, they can serve as a model for evaluating the interactions between the BBB and adjacent brain tissue and provide a platform for understanding the combined abilities of a new drug to overcome the BBB and its effect on brain tissue. In addition, such models are highly scalable and easier to manufacture and operate than microfluidic devices [143].

The efforts of vascularized BBB spheroids have also been reported. Using primary cells from humans [4,143,144], mice [145], and cells from both sources [146], capillary-like structures were formed in these models. Urich et al. [144] were the first to describe spontaneous self-assembly of commercial human cell lines of primary BMECs, primary pericytes, and primary astrocytes into a defined cellular structure that could recapitulate the complex arrangement of each cell type in the BBB structure. They adopted the Matrigel® and 3D hanging drop spheroid models to investigate the inherent association properties between these key cells. One of the most intriguing results of this study was the observation that the cells in the spheroids spontaneously self-assemble and reproduce the morphological arrangement of the different cell types in the BBB. The endothelial cells form an outer monolayer, and the astrocytes assemble in the center of the spheroids, while the pericytes line up in between, mediating an arrangement of three cell layers, suggesting a crucial role of pericytes in the assembly process. Overall, the results contributed to a better understanding of several key principles of cell-cell communication and interactions within the BBB [144]. Similar self-organizing phenomena have also been observed by Cho et al. [4]. They followed their method with slight modifications. They co-cultured primary human astrocytes and human brain vascular pericytes with two different brain endothelial cell types: primary hBMECs and immortalized human cerebral microvascular endothelial cell line D3 (hCMEC/D3) in the presence of VEGF-A. Since VEGF-A has been shown to promote vascular permeability by disrupting TJs and degrading the BBB, supplementing the culture medium with VEGF-A should be beneficial for BBB (neo)formation. They found that all cells in the co-culture interacted closely with each other and eventually self-assembled into a compact spheroid after 12 hours [4]. In addition, their results were consistent with the previously reported study, i.e., astrocytes mainly occupied the spheroid core, while both endothelial cell lines together with pericytes appeared to form a monolayer on the surface surrounding the spheroid [4,144]. Overall, the proposed BBB spheroid model could reproduce the properties and functions of the BBB characterized by high levels of tight/adherens junctions, efflux pumps, and transporters required to limit or regulate the influx of foreign molecules [4]. Also inspired by Urich et al.’s work [144], Nzou et al. [143] attempted to mimic the unique niche of human normal brain tissue more closely. They have created a 3D multicellular spheroid model of the BBB that demonstrated a functional formation of BBB. The charge selectivity of the formed BBB and its responses to conditional physiologic changes suggested that this spheroid model has the potential to discover novel therapeutics and evaluate the ability of such drug candidates to overcome the BBB [143].

Despite all the breakthroughs, these BBB spheroids lack an actual barrier between the “blood” and the “brain tissue,” making it impossible to measure TEER. Namely, in BBB spheroid models, blood vessels arise within a bulk of cells, without the inner tissue being covered with an endothelial lzyer [97,113].

### Microfluidic 3D models

Hemodynamics has a direct influence on the mechanical properties of the endothelium, and several pathologies are associated with disturbances in blood fluid dynamics, particularly in brain tissue. These dynamics are closely related to the tubular shape of blood vessels. Therefore, the presence of microvascular networks would provide an effective barrier between *in vitro* blood and the tissue, which is essential for the functionality of an *in vitro* BBB [97,147,148]. Recent advances in organ-on-a-chip technology have opened up the possibility of reconstructing the BBB microenvironment by incorporating various biomechanical cues into the constellation [149]. Unlike organoids (spheroid models), microfabrication-based models effectively create a tubular blood-tissue barrier, critical for modeling drug delivery [6,19,97,113,116]. Current dynamic 3D models with patterned vasculature are still in their infancy. Most studies focus on the simple fabrication of BMEC vessels and the evaluation of their properties. Nevertheless, they offer the unique advantage of studying molecules diffusing radially from a source with well-defined geometry, as well as directly imaging and monitoring BMECs as different molecules are applied to them. The diameter of the patterned vessels is much larger than the microvessels in the brain, and co-culture with direct contact is difficult or impossible in some of these models. In addition, there is no standardization of the models, as the vessel diameters and ECM composition vary greatly depending on the origin of the BMECs, and the throughput of these models is very low [6].

The assembly of vascular microfluidic 3D models is divided into two main categories: Organoid-like self-assembly and channel/mold assembly. The latter is more common in new perfused BBB platforms. In this approach, cells adhere to the surface of a channel or to the lumen of a biomaterial, which increases robustness and reproducibility compared to self-assembly models [97]. Among the various models that follow the channel/mold approach, is also the whole human-derived BBB platform (BBB chip), which combines human astrocytes, pericytes and endothelial cells [97,149]. In this microfluidic BBB platform, hBMECs cultured in the vascular channel under physiological shear stress formed an intact monolayer with TJs, while human brain vascular pericytes were cultured with human astrocytes on the other side of the porous membrane in the perivascular canal channel. More importantly, the human astrocytes embedded in the hydrogel showed a typical star-shaped morphology with the radial distribution of fine branching and their 3D cellular network. Moreover, quantitative analysis of gene expression of reactive gliosis markers, such as vimentin and lipocalin-2, confirmed that human astrocytes cultured in a 3D architecture in the BBB chip were more physiologically relevant than conventional 2D culture systems. Overall, the use of hBMECs in this BBB chip enabled the replication of BBB-specific endothelial features with stronger gene expression of junctional markers, membrane transporters and receptors, resulting in a tight barrier with permeability comparable to current tri-culture models. Moreover, this platform favored a 3D astrocytic network with reduced reactive gliosis and polarized AQP4 distribution. In addition, it accurately captured the distribution of 3D nanoparticles at the cellular level and demonstrated differential cellular uptake and penetration into the BBB through receptor-mediated transcytosis. Based on their results, the authors proposed the developed BBB chip as a complementary *in vitro* model to animal models for the prescreening of drug candidates for the treatment of neurological diseases [149].

Most models use compartmentalization in microfluidic channels to create the different cell layers or lumens. These layers can be formed either by encapsulation with hydrogel or by adhesion to the channel walls [97]. Thus, optimization of ECM composition and compliance is required to allow endothelial cell invasion and ECM degradation. At the same time, the hydrogel must also be structurally stable to allow long-term culture and perfusion of nutrients. It must also accommodate supporting cells such as pericytes and astrocytes within the gel to support the newly formed vasculature [6,150]. The importance of mimicking the vascular niche of the CNS for the successful establishment of an *in vitro* BBB model was also recognized by Lee et al. because BBB development occurs in three sequential steps: angiogenesis, differentiation, and maturation [150]. They developed a microfluidic *in vitro* BBB model that mimics CNS angiogenesis in order to maximize the physiological relevance of the human BBB. For this purpose, a mixture of hBMECs and pericytes was seeded on the surface of a fibrin gel containing astrocytes. In addition, another compartment containing fibroblasts was on the chip to provide a chemotactic gradient to support angiogenesis. In this model, all three types of primary human cells (hBMECs, astrocytes, and pericytes) exhibited *in vivo*-like 3D morphology, with direct cellular interactions occurring within the microfluidic channel. These observations confirmed the necessity of the angiogenic triculture system (brain endothelium interacting directly with pericytes and astrocytes) to attain essential BBB vessel phenotypes, such as minimal vessel diameter and maximal crossover expression. Moreover, lower vessel permeability was achieved in triculture than in monoculture. They also focused on the reconstitution of the functional efflux transporter system and demonstrated the higher efflux property and the outstanding effect of inhibitors in the triculture model after treatment with efflux transporter inhibitors [150]. Previously, Herland et al. [76] used a 3D model of the human BBB (3D BBB-on-a-chip) to reveal direct interactions between cells in the absence of an *in vitro* barrier (porous membrane). They constructed a 3D microfluidic model of a hollow human brain microvessel containing closely apposed human primary microvascular endothelial cells, pericytes, and astrocytes to analyze the contribution of each cell type to neurovascular responses to inflammatory stimuli. Studies were performed using the engineered microvessel with stratified endothelium in the presence or absence of primary human brain pericytes under the endothelium or primary human brain astrocytes in the surrounding collagen gel. This study design allowed them to investigate the ability of the simplified human BBB chip to identify the differential contributions of these supporting cells to the neuroinflammatory response. The developed 3D BBB-on-a-chip exhibited similar barrier permeability to that observed in other *in vitro* BBB models using non-human cells. When stimulated with the inflammatory trigger tumor necrosis factor-alpha, different secretion profiles for granulocyte colony-stimulating factor (G-CSF) and interleukin-6 (IL-6) were observed depending on the presence of astrocytes or pericytes. Both G-CSF and IL-6 are involved in neuroprotection and neuroactivation *in vivo*. Since the levels of these responses detected in the 3D BBB chip were significantly higher compared to static transwell BBB models, this 3D BBB chip has shown promise as a research tool to study human neurovascular function and inflammation *in vitro* and to identify physiological contributions of individual cell types [76].

Despite the progress in microfluidic BBB devices, these organ-on-a-chip devices are often too technically complex, require highly specialized setups and equipment, and are unable to detect temporal and spatial differences in the transport kinetics of substances that migrate across cellular barriers [6,75,97]. To overcome these shortcomings, Brown and his team [75] reported an easy-to-use 3D microfluidic platform for the human BBB (μHuB). They used a commercially available microfluidic device (SynVivo Inc.) as a scaffold, on which they cultured hCMEC/D3 and primary human astrocytes. To promote cell adhesion and proliferation, this commercial platform was initially coated with various basement membranes (rat tail collagen type 1, human fibronectin, and laminin). Within the μHuB-system, hCMEC/D3 monolayers sustained physiologically relevant shear stresses over a day and formed a complete inner lumen that resembled blood capillaries *in vivo*. These monolayers expressed phenotypic TJ markers (claudin-5 and ZO-1) whose expression increased after the presence of hemodynamically similar shear stress. Due to the transparent nature of the glass and polydimethylsiloxane, the model allowed direct monitoring of the barrier and associated transport in the presence of physiologically relevant shear conditions. The authors also demonstrated the modularity of the μHuB, which can be readily adapted for more complex co-culture experiments to further bridge the gap between existing tools for studying the human BBB and its underlying biology [75].

In contrast, organoid-like microfluidic models that self-assemble rely entirely on cell-cell recognition to form a network of blood vessels that perform angiogenesis *in vitro*. Vascularization is crucial for their growth and the development of a multicellular architecture [6,97]. Recently, some progress has been made in this field by incorporating endothelial cells into organoids. Although the overall vascular architecture is not controlled as in the vascularized channel/mold BBB models, it has proven to be robust and has been successfully used in representative microfluidic organoid models [97,151-153]. Based on the hypothesis that the co-culture arrangement could support the maturation and differentiation of human iPS cell-derived endothelial cells (iPSC-ECs) into BBB microvascular cells, Campisi et al. [151] designed a self-assembled 3D microvascular model of the human BBB. The proposed microfluidic system contained human iPSC-ECs, brain pericytes, and astrocytes. This multicellular culture was able to self-organize into vascular networks via vasculogenesis in fibrin gel, thus better mimicking neurovascular organization *in vivo*. The fibrin gel, which was held in a central chamber by surface tension, promoted self-organization of the perfused blood vessels. It also allowed all cell types to be in dynamic and direct contact with each other, resulting in a spontaneous arrangement into organoids. The BBB model was characterized by gene expression of membrane transporters, TJ and ECM proteins, all indicative of BBB maturation and microenvironment remodeling. Moreover, it exhibited perfusable and selective microvasculature, with the lower permeability than conventional *in vitro* models and similar to *in vivo* measurements in rat brain. Therefore, this proposed 3D human model of the self-assembled BBB is believed to be a reliable and valuable next-generation system that will advance the understanding of the neurovascular function and enable the preclinical development of effective CNS therapeutics [151].

Alternative strategies have also been proposed that do not fit into the ones aforementioned, but they generally focus on specific features or goals that require a specific design and layout strategy [97,154-156]. For example, the use of hollow fibers can produce highly controlled and well-defined vessels [97,154]. Hollow fiber-based design enables the construction of a flexible 3D BBB *in vitro* model that could allow for the systematic study of biological mechanisms with increasing levels of complexity. As has been shown, this approach can support the growth of human cells in 2D (defined here as monolayers on a surface) or 3D configurations (cells within a gel), as mono- or co-culture, and under static or dynamic conditions. Furthermore, the human astrocytes in the gel matrix exhibited a characteristic star-shaped morphology throughout the surrounding gel. Because this platform consisted of a hollow fiber populated with hBMECs and neuronal cells, it allowed the flexibility to change configurations to support mechanistic experiments of varying complexity without compromising the functionality of the BBB *in vitro* [154].

These advanced 3D BBB-on-a-chip systems are unique in their capabilities and, with further advances, are likely to provide important insights into transport mechanisms at the capillary level. However, it is unclear how to make direct measurements of permeability in these models due to the limited perfusion and complex, poorly defined geometry of the newly formed microvascular network [6].

## CONCLUSION

The BBB is a fundamental component of the CNS, as its functional and structural integrity is essential for maintaining the homeostasis of the brain microenvironment. Since deterioration of BBB function has been associated with the pathogenesis of neurological diseases, efforts have been made not only to develop effective therapeutics but also to better understand the biology and (patho)physiology of the BBB. Considering the directive to reduce animal testing, there is also a need to develop *in vitro* models to replace them. The use of *in vitro* BBB models for drug delivery and study design is not novel.

To date, these models have provided valuable information by serving as high-throughput complements to animal models. The transition from BMEC monocultures to multicellular BBB models with one or more additional NVU cell types has greatly expanded their potential beyond screening drug permeability to the study of molecular and cellular mechanisms underlying BBB physiology and disease. Current models vary widely in cost, technical requirements, recapitulated BBB aspects, and intended applications. Simpler models perform better in assessing quantitative parameters than biomimetic models, which are superior in phenotypic mimicry.

Future guidelines should incorporate the cellular knowledge gained from simpler models with consistent quantitative performance and apply it to complex models that still underperform in the defined gold standards of the field. Furthermore, the inclusion of physiological shear stress is critical to the representativeness of these models and should be included in future models. As knowledge of the BBB increases, the development of more accurate and complex *in vitro* systems will continue, particularly with regard to the phenotype and reproducibility of *in vitro* BBB models.
